# Roadwork: expertise at work building roads in the Maldives

**DOI:** 10.1111/1467-9655.13236

**Published:** 2020-03-23

**Authors:** Luke Heslop, Laura Jeffery

**Affiliations:** ^1^ London School of Economics and Political Science Houghton Street London WC2A 2AE UK; ^2^ University of Edinburgh 5.20 Chrystal Macmillan Building, 15a George Square Edinburgh EH8 9LD UK

## Abstract

This article engages critically with concepts of ‘skill’, ‘expertise’, and ‘capacity’ as they operate as markers of distinction and domination and shape migratory labour relations among road construction workers from across South Asia in the Maldives archipelago. The article examines roadwork at three levels: the professional biographies leading to ‘flexible specialization’ rather than technical expertise amongst Maldivian managers; the technical expertise and social incorporation of ‘skilled’ Sri Lankan supervisors; and the key material expertise of ‘non‐skilled’ Bangladeshi labourers in precarious employment. Whilst discussions of South Asian labour migration have been dominated by caste and class, this article argues that it is important to consider how the cultural production and understanding of concepts such as ‘expertise’, ‘capacity’, and ‘exposure’ at worksites can (also) become distinguishing factors in (hierarchical) migratory labour relations.

## Abstrait

En chantier : l'expertise au travail dans la construction de routes aux Maldives

Résumé

L'article examine de façon critique les concepts de « compétence », « expertise » et « capacité » comme marqueurs de distinction et de domination et facteurs d'organisation des relations de travail sur les chantiers routiers. Venus d'Asie du Sud et de tout l'archipel des Maldives, ces travailleurs sont étudiés à trois niveaux : les biographies professionnelles qui conduisent les cadres maldiviens de l'expertise technique à la « spécialisation flexible », l'expertise technique et l'intégration sociale des contremaîtres srilankais « qualifiés » et l'expertise matérielle essentielle des ouvriers bangladais, « non qualifiés » et précaires. Alors que les discussions sur les migrations de travail depuis l'Asie du Sud sont jusqu'ici dominées par les notions de caste et de classe, les auteurs avancent qu'il est important d'examiner la façon dont la production culturelle et la compréhension de concepts tels que « capacité », « expertise » et « exposition » sur les chantiers peuvent (aussi) devenir des facteurs distinctifs dans les relations au sein de la main‐d’œuvre (migrante).

The immigration queues at the Maldives’ international airport reveal a stark spectrum of human privilege and precarity. On the left, First and Business class priority passengers wait on a lush red carpet, a nod to the world's rich visiting the exclusive resorts that form the backbone of the Maldivian economy. This multi‐billion‐dollar industry is controlled almost entirely by the private sector: a combination of joint venture companies and conglomerates owned by a handful of Maldivian families. In the central queues, Maldivian nationals line up. To their right, the longest and slowest moving queues are dedicated to those newly arrived on working visas. This batch, referred to by the catch‐all ‘expatriate workers’, are documented and categorized as either ‘skilled’ or ‘non‐skilled’ (Saeed 2018: 22). Falling into one of these two categories carries implications of social standing and respectability, but ‘skill’ is also mapped onto precarity, whereby ‘non‐skilled’ equates to replaceable, exploitable, and invisible.

Starting with the uneven insertion of migrant workers into the layered Maldivian labour market, this article engages critically with concepts of ‘skill’, ‘expertise’, and ‘capacity’ as they operate as markers of distinction and domination, shaping migratory labour relations among construction workers in the Indian Ocean. Through an ethnography of roadwork, the article argues that hierarchical social relations at the worksite are mediated by both material and institutional interpretations of skill and expertise. Moreover, the article shows that these contextually derived interpretations are underpinned by professionalized narratives that both look back, to past ‘exposure’, and forward, to the development of worker ‘capacity’. To tell this story, the use of ‘professional biography’ is key. Expertise and skill, as we discuss it, represent a formalized category of employment indexical of precarity, but are also cultural ideas mapped onto the bodies of particular workers and used as a discursive trope among migrant labourers – albeit in different ways – as a form of distinction. This is brought into particularly sharp focus in the context of roadwork and road construction in the Indian Ocean, where skill sharing between institutions, individuals, and countries is a central component of project implementation. At a more general level, the article depicts the development of public goods through private means and illustrates how these specific circumstances reflect and reproduce the unequal experiences of workers within South Asia's ongoing experiment with liberalization.

The study of experts, expertise, and experience has a distinguished history in the social sciences, firstly in science studies (e.g. Collins & Evans 2002; Turner 2001; Wynne 1996; Yearley 2000) and a little later in anthropology (e.g. Boyer 2008; Good 2007; Harvey 2007; Harvey & Knox 2015; Holmes & Marcus 2008; Mosse 2011). In science studies, Collins and Evans (2002: 251, 260) distinguish between ‘certified expertise’ (formalized through qualifications) and ‘experience‐based expertise’ (in which experience is necessary but not sufficient for recognition as expert). Similarly, in anthropology, Boyer (2008: 39) links expertise to skill, competence, attention, and practice, highlighting tensions in the social recognition of ‘social‐institutional’ expertise (e.g. technocratic) compared to ‘experiential‐performative’ expertise (e.g. craft). In his work on apprenticeship in western India, Simpson noted that shipbuilders distinguished between their ‘easy’ craft, which they acquired through ‘non‐reflexive practice and repetition’, and the ‘difficult’ schooling or technical training, which they associated with ‘complicated manuals of regulations or numbers to read’ (2006: 161). In this and many other ethnographic contexts we see a hierarchy privileging work of the ‘mind’ (or ‘brain’) as superior to inferior ‘manual’ (or ‘physical’) labour (Simpson 2006: 162). We will return later to the distinction between working with one's ‘head’ and with one's ‘hands’. The salient point is that expertise is always interactional and ideological: ‘people become experts not simply by forming familiar – if asymmetrical – relationships with people and things, but rather by learning to communicate that familiarity from an authoritative angle’ (Carr 2010: 19), and ‘would‐be experts must continuously work to authenticate themselves as experts as well as to authenticate the objects of their expertise’ (Carr 2010: 21). ‘To the extent that practitioners are successful in establishing their expertise’, Carr notes, ‘they can create hierarchies and distinctions by determining the qualities, authenticity, or value of the objects within their purview’ (2010: 21‐2).

Engineers – archetypal experts in the literature on expertise (Harvey & Knox 2015; Mitchell 2002: 19‐53) – are key figures in Lévi‐Strauss's (1966) classification of scientific knowledge as either creative or technical. Writing on expertise in roadbuilding, Harvey and Knox (2015: 107) draw on this distinction, contrasting (creative) *bricoleurs*, who, ‘finding themselves in a specific material and intellectual environment, use the means at their disposal to tackle problems that present themselves’, with (technical) engineers, who stereotypically ‘work with a more gridded understanding that isolates a specific problem and sets about finding a solution through innovation and experimentation. For Derrida (1978 [1967]), however, if *bricolage* entails borrowing one's concepts, then logically every actor engages in *bricolage*, and thus engineers are simultaneously also *bricoleurs*. In her work on ‘neoliberalizing the self’, Urciuoli (2008: 215) notes that the idealized neoliberal employee is expected to demonstrate a combination of ‘hard’ technical skills and ‘soft’ (inter) personal skills. Harvey and Knox similarly suggest that an individual worker might combine ‘hard’ and ‘soft’ skills in the form of the ‘engineer‐*bricoleur*’ who recognizes ‘the social dynamics of technical relations’ and engages flexibly with ‘uneven, unruly, and unstable environments’ (2015: 17). The professional expertise of roadwork engineers, understood as ingenuity in conditions of constraint, derives ‘as much from their capacities as *bricoleurs* as from their ability to produce and maintain distinct, abstract conceptual repertoires’ (Harvey & Knox 2015: 107).

In this article, we explore how young road workers, after attending technical college to gain engineering qualifications, become *bricoleurs* by gaining experience in some of the various forms of expertise that need to be brought together in roadwork: social networking, management and supervision, operating heavy machinery, digging channels and laying pipes, making bricks, cooking asphalt, and preparing cement. Following Harvey's (2007: 1) conceptualization of ‘expertise’ in terms of external knowledge as a source of transformation in development and planning paradigms, we explore how the Maldives Road Development Corporation (MRDC)^1^ sought to overcome local shortcomings in capacity by bringing in external experts under an explicit mandate to share their expertise. We thus reveal how ‘engineer‐*bricoleurs*’ journey towards socially recognized expertise and flexible employability through a combination of technical qualification, material experience, and social interaction. We show that roadwork is powerfully infused with professionalized interpretations of skill acquired through previous exposure to expertise. In this way, our arguments about distinction and expertise in roadwork could apply across neoliberal workplaces globally.

MRDC recognized a lack of capacity in the Maldivian roadbuilding industry, and therefore outsourced contracts to private sector foreign companies which were given an explicit mandate to develop local capacity and transfer knowledge about road construction. Employed in a range of ‘skilled’ and ‘non‐skilled’ capacities, Sri Lankans working on the roads were thought to provide the ‘expertise’ from which MRDC managers were expected to learn. Our ethnographic material suggests, however, that many Maldivian MRDC managers were ‘job‐hoppers’ (Upadhya & Vasavi 2008: 24): rather than seeking technical roadwork expertise, they amassed resources and cultivated political and industry networks, developing ‘flexible specialization’ (Yanagisako 2018: 50) to prepare themselves for upward mobility into wider circuits of employment in the Maldives and beyond. Meanwhile, much of the roadwork itself was carried out by Bangladeshi men with precarious employment and immigration statuses, whose material expertise was crucial to the success of the project on the ground, but whose work was officially defined as ‘non‐skilled’.

By examining roadwork in the Maldives through the anthropological literature on skill and expertise, we also further a critical discussion on mediated global production circuits within South Asia (Barnes, Lal Das & Pratap 2015; Barrientos 2013; Carswell & De Neve 2013; De Neve 2019; Mezzadri 2016) by suggesting that the cultural production and understanding of concepts such as ‘expertise’, ‘capacity’, and ‘exposure’ at worksites become distinguishing factors in (hierarchical) migratory labour relations, whereas elsewhere in South Asia caste and class have dominated such discussions. In the outsourced contractor setting, notions of skill and capacity become powerful cultural idioms of distinction. This work then links personal professional biographies, or what Larson refers to as a ‘professional mobility projects’, with ‘wider processes of social stratification at work in the “Great Transformation”’ (1977: 68; see also Polanyi 1957). The potential for a migrant worker to better their situation through the labour market is historically constituted in no small part by the colonial project. Inherited notions of expertise, prestige, and status travel with migrant workers to new sites across South Asia. We interrogate the social life of work through the development of island infrastructure and the experiences of workers at three different scales of the construction process. Contracted Bangladeshi labourers shift sand and dig road foundations under highly controlled – sometimes unfree – circumstances, supervised by a Sri Lankan construction team working on outsourced projects for Maldivian clients. Our case study offers a rare focus on international labour migration from several places as they converge in one particular destination: the Maldives archipelago.

## The Maldives Road Development Corporation (MRDC)

It may seem curious to focus on road development schemes in the Republic of Maldives, an archipelagic state in the Indian Ocean where the national territory covers an area of 90,000 square kilometres, but only 300 square kilometres qualifies as land (Bremner 2016: 289); indeed, only two of the roads discussed link up to any other islands. The other roads discussed here cover narrow coralline streets with asphalt, ostensibly to improve circulation and the movement of people and things around discrete islands. Whilst roadbuilding schemes form the ethnographic context of our work, we zoom in to the everyday processes and people that bring these projects into fruition. The Maldives has a reported total resident population of 402,071 (National Bureau of Statistics, 2015: 15) dispersed across twenty Administrative Atolls (encompassing 118 inhabited Administrative Islands) and Non‐Administrative Islands (109 tourist resorts and 128 industrial islands and islands used for other purposes). The Maldives offers a particularly interesting land/seascape for the study of roadwork expertise owing not only to the archipelago's fragmented geography, but also to the republic's relatively recent and rapid entry into global infrastructure development markets (Pal 2018).

The Maldives was a British Protectorate from 1887 until 1965, governed locally by a Sultanate rather than by administrators based in London. As a Protectorate, infrastructure development, national planning, and the organization of public works in Maldives were not subject to the same regimes of expertise institutionalized through the colonial experience in neighbouring India and Sri Lanka. The first national public works took place in the early 1950s, as part of a modernization plan under Prime Minister Muhammad Amin Didi. It was ad hoc and small in scale, and something Maldivians were forced to do by the Sultan or an Island Chief (Maloney 1980: 201). Whilst people today express pride in Maldivian construction and craft – particularly in relation to ship‐building – the figure of the engineer held little social cachet. To be an engineer did not carry with it the occupational prestige that engineering acquired elsewhere in South Asia as an enduring legacy of colonial public planning. In addition, the few Maldivian development projects of the mid‐to‐late twentieth century were not moored in postcolonial nationalistic visions of a politically (re)imagined precolonial past (Seneviratne 1999; Tennekoon 1988; Woost 1993). Nor was infrastructure development institutionalized and infused with inherited colonial symbols of advancement and expertise (see also Chatterjee 2004). These projects focused on commercial fishing, post‐harvest technology, and banking reform moored in the liberalization ideology of connecting supposedly isolated economies to the global market.

Architectural and infrastructural expertise entered the Maldives primarily through the private sector, for the most part to develop and service an exclusive resort economy rather than to develop the ‘inhabited islands’, which often continued to lack electricity and water and sewage treatment facilities. Funding for public infrastructure projects, according to the Vice‐President of the Maldives National Chamber of Commerce and Industry, came ‘through the assistance of the international donor community’ (Latheef 2002: 252), thought to pave the way for private sector participation in Maldivian economic life more broadly. The central role of the government in enabling the participation of the private sector in capacity building and infrastructure development was outlined specifically in the 2002 Maldives National Development Plan, referred to as ‘Vision2020’. Vision2020 largely reflected the World Bank's imperative for Public‐Private Partnership, which gained consensus within development discourse in the early 1990s (Latheef 2002: 242). Nevertheless, state‐owned enterprises (SOEs) in the Maldives are thought to have maintained an ostensibly unfair competitive advantage vis‐à‐vis the private sector.

Responsibility for road construction and maintenance shifted from Island Chiefs to municipal services and, in 2010, to MRDC, an SOE established as a corporate profit‐seeking unit under the aegis of the then Construction Ministry (now Ministry of Housing and Infrastructure). It wasn't until the election of the Maldives Democratic Party in 2009 that the Maldives developed an official policy on how to ‘open up’ the country to global markets on a commercial basis. MRDC received projects from the government budget without having to go through a competitive tendering process. It also acted as local partner for foreign investment and joint ventures. According to MRDC's Managing Director, the corporation was a ‘true business’ that avoided the procedural ‘hurdles’ normally associated with government procurement processes.

Despite the energy behind the corporatization of a public service, there was – according to many who worked for MRDC – a distinct lack of ‘expertise’. MRDC's Managing Director reported that the corporation had suffered from a limited capacity to develop roads until an increase in capital support from the government in 2013, whereupon it invested around 149 million Maldivian rufiyaa (around US$8.5 million) in roadbuilding equipment and development projects. In 2014, the Ministry of Housing and Infrastructure and MRDC jointly initiated the Seven Island Road Project (SIRP). The selected seven islands were chosen ostensibly because they were relatively more populated economic hubs. The plan was to transform their roads from sandy tracks to asphalted roads suitable for motorized traffic, despite low numbers of vehicle use. Outsourcing to foreign companies was rationalized in terms of institutional development and an opportunity for MRDC to gain ‘exposure’ to the private sector and develop the expertise to take on similar projects alone in the future. A skill‐sharing ‘capacity development scheme’ was written into subcontracts.

This article draws on twelve months of fieldwork in Maldives in 2016‐17, when Heslop worked with a consultancy firm in the capital, Malé, and participated in roadbuilding projects undertaken by a Sri Lankan construction company working on several islands across the Maldives archipelago. Heslop engaged principally with Maldivian managers and Sri Lankan supervisors in English, Sinhala, and basic Dhivehi; he observed their interactions with Bangladeshi workers, but direct engagement with Bangladeshi workers was limited owing to linguistic constraints and his prior incorporation into the managerial/supervisory level of the work hierarchy. Ethnographic material is supplemented with interviews with consultants, contractors, and engineers in the Maldives, and with government officials, politicians, and CEOs of state corporations in Malé and Colombo. Those working on the roads in the Maldives engaged directly with a concept of capacity building, a ‘buzzword’ (Cornwall 2007) that emerged in development discourse in the late 1980s (Eade 1997) in response to the era of participatory development (Douglas‐Jones & Shaffner 2017), when the development industry promoted ‘partnerships’ and ‘dialogue’ as opposed to top‐down strategies (Linnell 2003).^2^ Sri Lankan supervisors employed by the firm subcontracted by MRDC used the Sinhala word *dharanaya* (capacity). One might be told on the roadside: *obage dharana hakiyawa pramanāwath natha* (your capacity is not enough). Rather than being about qualifications, this generally refers to someone's ability to hold something in their head as opposed to in their hands. Inversely, if someone was skilled with a tool or machinery, they were generally referred to as *dhaksha* (clever), which in other contexts is a capacity of the mind. In Divehi, several phrases approximate to the concept of capacity building: *gaabiliyathukan ithurukurun* (qualification increasing), *hunaruverikan ithurukurun* (skills increasing; the phrase favoured in workshops and training events), and *heyluntherikan ithurukurun* (awareness increasing; the only one of the three phrases that refers to a development of the mind).

MRDC managers used the English word ‘capacity’ interchangeably both to refer to technical ability and the attainment of a formal qualification and as a signifier of the potential to become more skilled if given the opportunity. The latter, a personal inflection of the term, suggests that someone believes themselves to be capable of something but at the current moment is found wanting in some regard. For an aspiring engineer or a road company, developing capacity involves imagining potential futures and parsing out what a path to achieving this might look like. At an institutional level, the concepts of capacity and capacity building were used to quantify the scale of operations that could be carried out: the number of projects, the size of a project, the number of employees, and so forth. MRDC and its managers couched capacity building in terms of expertise to design and build, but they also saw the mediation of resources, maximizing of status, and holding office as skillsets cultivated in a private construction economy. A lack of capacity is at the heart of subcontracting, but there is a disconnect between the skills that MRDC claimed to lack and those its employees sought to develop. Whilst there was professed to be some sort of capacity building, loosely connoting skills and expertise transfer in the industry, it was difficult to see where industry expectations of capacity building might be taking place. In the following sections of this article we illustrate the concepts and processes of ‘exposure’, ‘skill‐share’, and ‘capacity development’ ethnographically by exploring the social life of roadwork through professional differentiation of workers at three different levels of the roadbuilding process: the cultivation of ‘flexible specialization’ rather than technical expertise amongst Maldivian MRDC managers; the technical expertise and social incorporation of ‘skilled’ Sri Lankan supervisors; and the key material expertise of ‘non‐skilled’ Bangladeshi labourers in precarious employment.

## Maldivian managers: ‘flexible specialization’ and a ‘technical guy’

Shameem, a young Maldivian man, found employment with the Public Works Service (PWS) after completing his A‐levels, and was later invited to go to Sri Lanka to undertake a training course on roadside asphalt laboratory work. His relatively short career with the PWS came to an end when the newly elected Maldives Democratic Party offered him a redundancy pay‐off, with which Shameem bought a vehicle for use as a private taxi. When the Chinese subsidiary Jiangsu Technology and Engineering Group (J‐TEG) arrived to build a link road across Laamu Atoll (see Heslop & Jeffery n.d.), one of the managers hired Shameem for the duration of the project. Shameem shadowed the Chinese manager at the planning stage, befriending the Chinese workers. His connections were evident in the fact that he had access to the construction group's otherwise restricted private accommodation, a huge complex made almost entirely of metal freight containers that the company had used to ship equipment. The disturbingly flat‐pack‐style accommodation units signalled impermanence. The offices had holes cut in the sides to create makeshift doors and windows. The complex was designed to be self‐sufficient, with a vegetable garden, a water purifier, and a livestock area, as well as a basketball court. Some Chinese workers remained on site even after the road was complete because responsibility was not handed over to the MRDC until the grand opening.

Shameem referred to his time with J‐TEG as an internship, an opportunity to gain exposure. He admired the management discipline of the Chinese contractors, impressed specifically that they didn't let the labourers out of the compound other than to work. When his ‘internship’ with J‐TEG came to an end, Shameem took up a construction job on a private island that was being turned into a resort. Here, he got first‐hand experience operating larger construction machinery and working with electrical wiring. He quickly got bored on the island, however, which was only about 400 metres long. Returning home to Laamu, he attributed his ability to secure a job as an MRDC supervisor to his exposure to the road development process through his association with J‐TEG, not to mention the good relations that he had developed with them, and his hands‐on experience with construction machinery on the private island.

Shameen assumed that when the maintenance of the link road was officially handed over to MRDC, the road would be ‘his’. He also assumed that the MRDC would inherit, purchase, or otherwise acquire J‐TEG's vehicles, which would mean that he would have *de facto* control over a great deal of construction equipment that he could manage and use to his own advantage. Shameem went from being a worker in the PWS, doing the bidding of the municipal council, to being a supervisor in MRDC, a profit‐seeking SOE that the municipal council paid commercial rates as an incentive to undertake infrastructural work in the public interest. In the process, he had also acquired a vehicle to use as a private taxi. Shameem framed his expertise in terms of the exposure that he had gained in the industry, which came about through relationships with managers and bosses more than through exposure to technical processes. Shameem acquired some technical know‐how on the resort island, but he considered that to become a successful roadman it was more important to sit on assets and cultivate the ability to mix with different kinds of people, so his interest in developing his technical capabilities waned as he became what Harvey and Knox would describe as an ‘engineer‐*bricoleur*’. Through his dealings, he gained transferable skills, but not what could be described as sector‐specific expertise.

Shameem was not alone in his focus on control over construction assets and budding leverage in local politics as an interpretation of what it means to be skilful, develop capacity, and ‘do well’ in the road business. Abdulla, the MRDC manager in Addu, the southernmost atoll in the Maldives, had a similar approach. He measured his success and skill in the road development industry by the amount of unused equipment and materials he could amass in ‘his’ casting yard. Interlocking bricks, piled together neatly in blocks bound with plastic cable ties, sat unused. The bricks had been paid for by South Asian Association for Regional Cooperation (SAARC) countries, intended to build local roads and a large conference centre in which to host the 2011 SAARC summit. Either too many bricks had been bought, or, perhaps more likely, too few bricks had been used. In any case, the much‐coveted Emirati interlocking bricks, the sort used for roads in Malé, were now at the disposal of MRDC, and, by extension, the savvy local manager. They would not find their way to a road until a high price was paid. MRDC's control over bricks paid for with donated money was one thing, but island councillors also complained that they could not get the corporation to do any work on road maintenance whatsoever because central government would not give the city council enough budgetary support to pay MRDC's seemingly overpriced service fees and material costs, despite it being a government‐owned company.

In the few years that Abdulla had worked for MRDC, he had used his position astutely to his advantage. Before travelling to other countries – India, Sri Lanka, Thailand – he had managed to make personal contact with road construction companies, resulting in being met from the airport, transported, and accommodated during his stay. This was despite him not travelling abroad in any official capacity. ‘I have all of the numbers’, he boasted, ‘because I sit on the bidding committee that awards the contracts to outside companies’. He perceived this practice as commendable extracurricular dedication to the road industry because it evinced his commitment to learning about road engineering and developing international contacts in the industry through his own efforts. He was also proud of having successfully changed his daughter's Presidential scholarship from medicine to engineering (which is no easy feat). Addu's particularly underfunded municipal council and the overall lack of materials and machinery makes the MRDC manager an influential figure on the atoll. Abdulla was particularly skilled in taking advantage of the assets under his authority and navigating the politics of construction, whether a sinecure on a committee or a paver and a pile of bricks in the casting yard.

Abdulla's assistant Latiff had been working on the roads in Addu ever since many of them had been built and maintained under the government company Southern Utilities Ltd. Like Shameem, Latiff had gained experience through foreign construction companies: firstly, the Danish company Hoegaart Plc during the construction of the Addu Link Road, and later with the Sri Lankan Road Development Authority, which developed the island roads in advance of the SAARC summit. Whereas Abdulla was a political operator, however, Latiff was what people in the infrastructure industry in the Maldives referred to as a ‘technical guy’. While his manager Abdulla was negotiating trips to Thailand and Sri Lanka with suppliers and contractors, Latiff spent his time in the casting yard trying to make his own bricks modelled on the Dubai design but using local materials at a lower cost. Latiff's bricks, as they were known, were used for local projects such as schools. Latiff had learned how to ‘cook’ asphalt in smaller batches, so the larger industrial machinery did not need to be fired up, thus reducing production costs. He cooked asphalt in old barrels that had been cut in half and laid on their sides like barbecues. By ‘cooking’ his customized smaller quantities of asphalt, smaller jobs would not become disproportionately and prohibitively expensive. Latiff could go to the roadside and fix holes or other damage as it was reported to him. Political and technical expertise necessarily coexist in the road construction industry, with some personnel plugged into international networks of construction expertise, and others on site delivering the goods themselves.

## Sri Lankan supervisors: technical expertise and social incorporation

In 2014, as noted above, MRDC with the Ministry of Housing and Infrastructure initiated the SIRP to develop roads on seven of the more populous islands. The contract was awarded to the Sri Lankan construction company Sierra Plc. Employed in a range of ‘skilled’ and ‘non‐skilled’ capacities, Sri Lankans working on the roads were thought to provide the ‘expertise’ from which MRDC managers were expected to learn. In fact, as part of the contract, Sierra were given an explicit mandate to develop local capacity and transfer knowledge about road construction to MRDC employees.

During fieldwork, the SIRP at the Kulhudhaffushi site was in the foundation stage: trenches were being dug and the concrete structures for the cable junctions were being laid. Implementation was the responsibility of a handful of young men from Sri Lanka employed by Sierra. The Sierra supervisors remained on site throughout a project's life cycle, which could amount to several years, with infrequent rotations to other islands. Those with basic civil engineering qualifications from technical colleges were supervisors classified as ‘skilled workers’, as were those who might not be supervisors but had a licence to operate a heavy goods vehicle of some sort. Those without such qualifications were categorically ‘non‐skilled’ and would do manual labour alongside Bangladeshi workers. ‘Non‐skilled’ Sri Lankans tended to speak more Divehi and became *de facto* supervisors to the Bangladeshi workers. Here, ‘non‐skilled’ Sri Lanka workers assumed a role akin to that of foreman, asserting a superior position vis‐à‐vis Bangladeshi labourers, who occupied the lowest rung in the workplace hierarchy. A grasp of Divehi to communicate with Bangladeshi workers made ‘non‐skilled’ Sri Lankans a particular kind of labourer on whom the ‘skilled’ supervisors depended. Asserting a superior position involved announcing breaks and handing out basic equipment like brooms and simple tools. Consider, by way of contrast, the role of the foreman in Elisabeth Schober's exposition of a South Korean shipyard in the Philippines, where a disciplinary regime subjects the Filipino workforce to military‐style drills (Schober 2018: 205). Schober demonstrates how an inherited military working culture, in which subordinates must be ultimately controlled, is enacted on Filipino workers. Unlike the Korean foremen depicted by Schober, Sri Lankan ‘non‐skilled’ workers similarly inhabiting the role of foremen were comparatively gentle, in fact largely sympathetic; many pitied the Bangladeshi workers and the assertion of hierarchy was relatively subtle. They were less invested in instilling any sort of ‘Sri Lankan working culture’ on the Bangladeshi labour force.

Regardless of a Sri Lankan employee's position in the labour hierarchy, Sierra was a well‐known company and the work Sri Lankan employees did in the Maldives was a source of prestige for them among their families back home. As men working overseas for a Sri Lankan company, the workers were not subject to a moralized discourse around out‐migration in the same way women have been in the context of domestic migration from Sri Lanka to the Gulf (Frantz 2013; Gamburd 2000, 2011; Spencer 2003). Sri Lankan ‘skilled’ workers distinguished themselves as migrant workers in the global labour industry by referring to themselves as ‘technical guys’, and saw taking on managerial tasks and organizing labour as a process of personal betterment. They were well incorporated into local social affairs and invited to participate in events such as weddings and festivals. However, some Sri Lankan supervisors were more adept than others at immersing themselves within island life.

Janaka, a Sri Lankan supervisor in his mid‐twenties, managed the construction work. He described his arrival on the island with a sense of personal achievement and adventure: he came to the site alone, without any equipment, and had to establish a relationship with local authorities, conduct surveys alone, and begin the preparatory groundwork. His proudest achievement, he joked, was that he had learned to cook. Unlike in Harvey and Knox's account of Peruvian roads, there were no specialized ‘Social Relations’ departments dedicated to integrating locals into the ‘common project’ here (Harvey & Knox 2015: 201). It was people like Janaka who had to manage the development of the road, and simultaneously people's expectations of the development process. The Kulhudhaffushi component of the SIRP was Janaka's first site supervising job. Despite his young age, he seemed to have earned respect from the other workers, who appeared to listen to him and follow his instructions, which he attributed to working hard and treating people nicely. In contemporary West Bengal, migrant workers are courted politely at the point of recruitment but spoken of in discriminatory terms in private (Rogaly *et al*. 2003: 293). By contrast, Janaka was sympathetic to the plight of the Bangladeshi workers employed on the site: he did not make inappropriate jokes, did not speak harshly or aggressively, and was not, according to his Sri Lankan colleagues, *sari* (spicy, aggressive).

Janaka worked hands‐on at the roadside and non‐stop throughout the day in the JCB, which people agreed he operated expertly. Operating machinery is a salient marker of being a ‘skilled worker’ with status in the industrial hierarchy. Similar narratives of status deriving from operating machinery have been noted in the BSP steelworks, where machine operators were likened to Brahmins or Bengalis (Parry 2003: 232). There were few, if any, verbal ‘communicative events’ in which expertise, or the performance of expertise, could have been at stake (Carr 2010; cf. Agha 2007). Sri Lankan workers gave instructions and commands to the Bangladeshi workers in crude pidgin Divehi, but they could not communicate complex or detailed instructions; on rare occasions, a Sri Lankan supervisor might explain something in English to a Maldivian MRDC employee who could then explain it to the Bangladeshi workers in Divehi. Bengali, Sinhala, and basic Divehi, spoken as a second or third language, were all in operation at the worksite.

Having already acquired technical expertise, Janaka hoped to develop his management skills: in other words, to become an ‘engineer‐*bricoleur*’ (in Harvey and Knox's terminology). He was at the forefront of daily dealings and negotiations with island residents about the quality of the road, with the local council about interruptions to the work, and with the MRDC managers who regularly wanted to borrow Sierra's equipment and charge a premium to loan Sierra theirs, which Sierra occasionally paid. Janaka had to manage people below him and answer to those above; nevertheless – unlike the Maldivians, whose career progression depended on job‐hopping, and the Bangladeshi labourers lacking opportunities for career progression – he envisioned a clear path up through the company hierarchy as a direct result of his sojourn in the Maldives (cf. Yanagisako 2018). In the case of Janaka and Shameem, as with many of the managers and supervisors Heslop worked with on construction sites and in container‐like site offices across the archipelago, talk of ‘exposure’ and personal capacity development in the workplace was the production of an immeasurable and intangible and inalienable property. Notably, as Larson describes in reference to *professionalism*, ‘the producers themselves have to be produced if their products or commodities are to be given a distinctive form’ (1977: 14). Outlined above are aspects of working practice that gave supervisors and managers like Janaka and Shameem a recognizably distinct supervisory/managerial product. A socially recognized qualification must also be supplemented through social interaction at the worksite in dealings with employees, peers, and clients. Referring to oneself as a ‘technical guy’ aided the managers’ ability to navigate the local politics of construction as well as, importantly for Sri Lankans working on the projects, distinguish themselves from ‘non‐skilled’ workers.

## Bangladeshi labourers: material expertise and contracted precarity

The story of ‘flexible specialization’ amongst Maldivian MRDC managers and the technical expertise of the ‘skilled’ Sri Lankan supervisors, however, captures only part of roadwork in the Maldives. Migrant workers comprise almost half of the archipelago's working population.^3^ A large proportion of migrant workers are employed in the tourist sector, which is the mainstay of the Maldivian economy. Masseurs, front‐of‐house staff, middle managers, doctors, and teachers arrive from across Asia as ‘skilled’ workers. But many, particularly from Bangladesh, Nepal, and the Philippines, arrive as ‘non‐skilled’ labour to work in cafés in Malé, local shops and barbers on islands up and down the archipelago, or out of sight on the agricultural islands, fish canning plants, and export processing zones often owned by Maldivian resort tycoons. The most precarious and least respected work, however, is construction. In this industry, ‘non‐skilled’ migrant labourers, predominantly men from Bangladesh, are brought to the Maldives to work for the companies responsible for the delivery of bridges, causeways, and link roads connecting islands within an atoll.^4^

Socially and culturally produced notions of skill and expertise in contemporary labour hierarchies are irremovable in their analyses from a history of debt bondage, bonded labour, and migration in South Asia (Brass 1990; Carswell & De Neve 2013). Outsourced labour contracting in the Maldivian construction industry encapsulates mediated employment relations across South Asia, a growing phenomenon which may be the root cause of precarity for many (De Neve 2019). Bangladeshi men, employed through agencies and individual brokers, are vulnerable to exploitation and work in relatively ‘unfree’ circumstances (see Barrientos, Kothari & Phillips 2013: 1039; cf. Brass 2004; 2009). Like Bangladeshi labourers elsewhere (see Shah *et al*. 2017; Standing 1981: 200), their mobility is heavily restricted, they have little formal integration into Maldivian bureaucracy, and their exploitability is compounded by almost complete exclusion from Maldivian social life (at least on the islands where Heslop worked).^5^ The bureaucracy of local sponsorship that keeps workers in the Maldives is a variant of the *kafala* system that underpins and regulates migration in the Middle East and produces conditions of ‘contract slavery’ (Gardner 2010: 58). Entry regulations bind employees to specific employers in a relationship of dependency, while restricted inter‐island mobility compounds workers’ fixity to the islands and the worksite. How Bangladeshi migrant workers are drawn to these labour arrangements is an area in which our data is thin, in part owing to the sensitivity of pursuing such questions from the labour site. Labour brokers from Bangladesh operating as outsourced contracting agencies worked directly with the construction firms. In other cases, Bangladeshi labourers living and working on the islands already could be hired on site as day labourers. The deep levels of indebtedness of the worker to the brokering agency was an issue of concern and speculation among the Sri Lankan workers; it was thought that in a five‐year working contract, Bangladeshi labourers would be paid for three, whilst the other two years of work would be pocketed by the broker. Bangladeshi labourers who could speak to Heslop in English were promptly ridiculed by the Sri Lankans, by way of discouragement. When Sri Lankans derided Bangladeshi English‐speakers, it was couched in terms of averting the embarrassment that would come should their own language proficiency in English be found wanting.

Processes to cut company costs, ensure cheap and flexible labour, and redefine labour as ‘non‐skilled’ can be observed in diverse work arenas globally: from fruit farms in Southern Africa (Bolt 2013) to shipyards in San Francisco (Blum 2000). In the Maldives, the hands‐on labour at the roadside is almost exclusively done by Bangladeshi workers, who are deskilled by definition by being officially categorized as ‘non‐skilled’. But roadbuilding on small islands is rarely executed as planned. Unforeseen issues arise that require improvised on‐site solutions, revealing the ‘indispensable role of practical knowledge, informal processes, and improvisation in the face of unpredictability’ (Scott 1998: 6). It is in the working through of problems at the roadside that the managerial hierarchies and the ‘hierarchies of knowledge and practice’ (Lambek 1993) in the road construction industry become blurred. Bangladeshi workers with their feet in the sand manipulate materials to find solutions, while the client (MRDC) ostensibly in charge of the project remains largely unaware of the processes going on at the roadside. Thus, ‘formal schemes of order are untenable without some elements of the practical knowledge that they tend to dismiss’ (Scott 1998: 7).

Before a single batch of asphalt can be made, the settlement tanks and cable junctions need to be laid under the trace of the road. The workers break up the ground using pickaxes and farming spades with actions reminiscent of digging an irrigation channel in a paddy field. A short arm crane fitted to the back of a JCB is used to install the cable junctions. Janaka pushes down the hydraulic legs and lifts the concrete box by looping a thick strap through it and connecting it to the teeth of the crane. The heavy precast concrete is raised and swung towards the Bangladeshi workers with callused hands, who guide it carefully into the hole that they have dug and in which they stand barefoot until the last moment when the concrete falls into place. When it is in position, they start to connect the grey PVC pipes into the three holes that lead in and out. The holes aren't a perfect fit for the pipes: some must be hit to fit in place, while the gaps left by smaller pipes are filled with stones found nearby and quickly cemented in position.

The cement for this quick fix is mixed in a small bucket carried around by an agile Bangladeshi worker who jumps in as soon as the pipes are fed through. There is no continuity in terms of the sand used; his measurements are entirely judged by sight and texture. As he quickly selects and collects appropriate stones for aggregate, he scoops up quantities of sand by sight and adds it to the bucket with water and cement as required. This is slap‐dash work but could not be described as inexpert or ‘unskilled’. Like forcing the PVC pipes into the cable junction holes, laying the pipes in the channels requires a degree of improvisation and measured force. In places the pipes need to be squeezed into the channel or cut and reconnected to other bits of pipe to make them more amenable to bend around an immovable object in the ground. Problems encountered at the roadside are worked out on the spot, where quick ad hoc solutions are desirable so as to press on with the work. There can be an awkward relationship between what different categories of the road crew know about the task and what they know about the materials. For example, a worker with his feet in the ground might be best placed to perceive how much a pipe might be forced to bend without breaking. The Sri Lankan supervisors would evaluate the work of Bangladeshi labourers at the end of each day. When the construction crew worked into the evening, large spotlights would shine into the channels that had been dug and the day's work was observed. The supervisors were primarily concerned that the pipes connected and the cable junctions were lined up rather than with teaching or learning in any performed or formal sense. Alongside the supervisors, shop owners along the roadside, people from the island council, and a representative from the TV company might come along to scrutinize the work of the Bangladeshi labourers, normally with the express concern that something already laid in the ground and belonging to them may have been taken or broken.

The official definition of the work done by Bangladeshi labourers as ‘non‐skilled’ does not recognize their material skills in the manipulation of matter in the ground, the use of tools, the ability to calculate weights and measures, in instinctive selection of stones for aggregate, and the ability to cut a PVC pipe so that it can be bent around an obstacle in the earth. In much the same way in India, Prasad‐Aleyamma emphasizes how port construction workers’ ability to ‘put in long hours of arduous labour, to grope in the mud, climb up walls, cart stones around and to hang down from tall machines’ makes ‘unskilled’ a nebulous term (2017: 183). In the case of Bangladeshis and Sri Lankans toiling in the Maldives, though ‘unskilled’ work is clearly skilled, the categorization of ‘skilled and ‘unskilled’ labour has significance beyond the wages paid for such work. For Sri Lankans in particular, being classified as a ‘skilled’ worker is an important mark of social distinction, a distinction (re‐)enforced with the self‐referential term ‘technical guy’. A supervisor lays out what is to be done, following a cut‐and‐paste format of South Asian Aggregate‐Base‐Course (ABC) road design, and communicates this ‘with familiarity from an authoritative angle’ (Carr 2010: 19). The skills and expertise of carrying out the task lies with the Bangladeshi workers. Theirs is an adept and craft‐like skill learned through practice rather than at a technical college. In roadwork, we see a clear split between what Boyer refers to as ‘experiential‐performative and social‐institutional poles of skilled knowing and doing’ (2008: 39). Being ‘technical’ – having attended a technical college and dealing in technocratic practices – detaches skill and capacity from the body, where it is located in craft‐like form among Bangladeshi labourers, and recasts it in the form of *expertise*, whereby it is socially validated and transposed onto processes and procedures.

Forms of financial remuneration mirror the relationship between skill and expertise, processes and bodies. In her discussion on the cultural politics of wages in Kerala, Prasad‐Aleyamma emphasizes how they reveal the politics of labour that both shape and are shaped by geographical relationships (2017: 165). The circulation of money through wage practices lays bare the social relations of global capital in worksites (Prasad‐Aleyamma 2017; see also Bolt 2014; Heslop 2016). In the Maldivian context, money payments and the various currencies they are made in situate workers in particular positions of precarity and locate them in broader networks of financial circulation. For example, the Sri Lankan company Sierra received payment in US dollars (initially loaned to the Maldives National Bank by the Bank of Ceylon), and paid its Sri Lankan employees in Sri Lankan rupees directly to their Sri Lankan bank accounts. Many Sri Lankan supervisors and managers, while in the Maldives, would not acquire any Maldivian rufiyaa at all; even the internet data for their mobile phones was provided by Sierra. By contrast, the Bangladeshi workers were paid cash in local currency, Maldivian rufiyaa, which they would almost immediately exchange for US dollars to remit via a mobile money transfer. Few had mobile phones with internet capacity, and those who did would gather around public Wifi spots on the more developed islands to make use of the network's free 15‐minute allowance. Like Bangladeshi workers elsewhere (see Breman 2003; Shah *et al*. 2017), Bangladeshi workers in the Maldives have to work for several years before paying off the brokers who mediated their initial employment; it is not unheard of for them to have wages withheld and even be deported without yet having been paid at all. As labouring bodies who occupied the lowest rung in the workplace hierarchy, Bangladeshis had cash in foreign currency put in their hands, whereas the financial remuneration of the ostensibly more ‘skilled’ salaried Sri Lankan workers was detached from the body and channelled through international financial processes. The social relations of wage payment among migrant workers not only renders the institutional privilege of ‘skill’ ethnographically observable, but also demonstrates how the ‘non‐skilled’ construction labourer is exposed to predatory intermediaries, who reinforce the conditions of their precarity. While Sri Lankan and Maldivian salaried employees gain ‘exposure’, Bangladeshi contracted labourers become exposed.^6^

## Conclusion

Workers’ opportunities in the labour market vary. In the infrastructure world, this is in no small part connected to the structure of finance within projects. Loan agreements bring with them agreements about labour opportunities for particular populations. Sierra was awarded the SIRP as a contractor finance arrangement with agreed finance from the Bank of Ceylon. This observation raises a further question about the extent to which these national distinctions in turn are underpinned not only by colonial structures but also by the current status of different national states with respect to the capital funding of these infrastructure projects. This is not a uniform picture, however: while a Sri Lankan firm benefited from Sri Lankan financing, Shameem managed to cultivate opportunities and develop ‘capacity’ by aligning with a project funded by the Chinese government and similarly outsourced to a Chinese subsidiary company (J‐TEG).

While Maldivians and Sri Lankans engaged in roadwork have opportunities to develop ‘capacity’ and become exposed to ideas of management, governance, and discipline, there is little about liberalized and outsourced construction work that could be read along the lines of an ‘enterprise culture’ for the Bangladeshi workers (cf. De Neve 2019; Gooptu 2013). For them, the outsourced contract labour economy promised them work (if they were lucky) and minimal pay (if they were lucky). In the outsourced contractor setting, notions of skill and capacity become powerful cultural idioms of distinction and domination. For Bangladeshis, skill and capacity are situated in the body as qualities not acknowledged on industry terms, hence ‘unskilled’/‘non‐skilled’. For Maldivian managers and ‘skilled’ Sri Lankan ‘technical guys’, skill and capacity are transformed into expertise and revalorized as a ‘social‐institutional way of knowing and doing’ (Boyer 2008: 39). Critiquing a stark distinction between technical engineers and creative *bricoleurs* (Lévi‐Strauss 1966), we show that workers seek to combine ‘hard’ technical and ‘soft’ interpersonal skills (Urciuoli 2008) and to become ‘engineer‐*bricoleurs*’ (Harvey & Knox 2015) who are flexible and highly employable. Moreover, our research suggests that recognition as ‘skilled’ is also a social‐institutional way of *being* that can be correspondingly mapped onto a relatively less precarious situation, but also affords a new language through which migrant workers differentiate amongst one another. What our ethnography brings out in particular is the relationship between the technical expertise (and social incorporation) of Sri Lankan supervisors and the material expertise (and social exclusion) of Bangladeshi labourers. Furthermore, it illustrates how the opportunities to acquire skills in *bricolage* that characterize the ‘capacities’ of the engineers are not available to the Bangladeshi workforce, whose capacities and expertise are limited by their formal categorization into the workforce.

We have also shown that the uneven insertion of migrant workers into the layered Maldivian labour market reflects and reproduces the unequal experiences of workers within South Asia's ongoing experiment with liberalization. While this fragmentation of the workforce is infused with professionalized interpretations of expertise and skill acquired through exposure to the ‘private sector’, and whether this exposure is framed positively or negatively, such arrangements might also echo colonial structures of labour relations. Whereas established understandings see caste and class as central to the social distinctions and discrimination characteristic of migratory workforces in South Asia, we have argued a case from the Maldives that it is also important to attend to the ways in which professional, skilled, and unskilled labour are differentiated and enacted. It is plausible that new notions of skill and expertise and professional differentiations reinforce class and caste position. Across worksites globally, idealized neoliberal employees (Urciuoli 2008) engage in processes of professional distinction, and work to establish themselves as would‐be experts (Carr 2010). We have suggested that in the Maldives, the cultural production and understanding of concepts such as ‘expertise’, ‘capacity’, and ‘exposure’ not only capture this process, but also become distinguishing factors in migratory labour relations.

## NOTES

1 MRDC was replaced by the state‐owned Road Development Corporation (RDC) by Presidential decree in June 2019.

2 The language of capacity had emerged much earlier in South Asia, however, as a colonial preoccupation with why peasant farmers lacked commitment to work in industrial settings (De Neve 2019; Parry 1999: ix).

3
https://minivannewsarchive.com/business/half-of-workers-in-maldives-are-foreigners-economic-development-minister-92748 (accessed 7 February 2020).

4 Statistics on ‘non‐skilled’ workers are unreliable as many enter the country via unofficial routes.

5 This degree of exclusion is not uniform across the Maldives archipelago: Saeed reports from Gaaf Alif Atoll that Bangladeshi workers might settle, learn local dialects, marry locals, start families, and ‘know more about the goings on in the islands than locals because of their position engaging with locals and contractors (foreign and local‐foreign) who assign them work’ (2018: 39). A continuing theme on the islands visited by Saeed and Heslop alike was that Maldivians would not be employed to do the manual labour undertaken by Bangladeshi migrant workers.

6 This is a story particular to the construction field. There are Bangladeshi workers who would claim that coming to the Maldives and working on an inhabited island is far more preferable than working on an agricultural island or in an export processing zone, and many would prefer to do either of these within the Maldives than have to work in the Gulf. The notion of professional exposure – discussed by Maldivians and Sri Lankans with a positive inflection – was not readily observable in the daily activities of Bangladeshi workers.
